# *In vitro* effects of Apixaban on 5 different cancer cell lines

**DOI:** 10.1371/journal.pone.0185035

**Published:** 2017-10-12

**Authors:** Luigina Guasti, Alessandro Squizzato, Paola Moretto, Davide Vigetti, Walter Ageno, Francesco Dentali, Andrea M. Maresca, Leonardo Campiotti, Anna M. Grandi, Alberto Passi

**Affiliations:** Department of Medicine and Surgery, University of Insubria, Varese, Italy; University of South Alabama Mitchell Cancer Institute, UNITED STATES

## Abstract

**Background:**

Cancer is associated with hypercoagulability. However, several data suggest that anticoagulant drugs may have an effect on tumor development and progression mediated by both coagulation dependent processes and non-coagulation dependent processes. Therefore, we investigated the *in vitro* effects of Apixaban on cell proliferation, mortality, cell migration, gene expression and matrix metalloproteinase in 5 different cancer cell lines.

**Methods:**

The following cancer cell lines, and 2 normal fibroblast cultures (lung and dermal fibroblasts), were studied: OVCAR3 (ovarian cancer), MDA MB 231 (breast cancer), CaCO-2 (colon cancer), LNCaP (prostate cancer) and U937 (histiocytic lymphoma). Proliferation and cell mortality were assessed in control cells and Apixaban treated cultures (dose from 0.1 to 5 μg/ml, 0 to 96-h). Necrosis/Apoptosis (fluorescence microscopy), cell migration (24-h after scratch test), matrix metalloproteinase (MMP) activity and mRNA expression (RT PCR) of p16, p21, p53 and HAS were also assessed.

**Results:**

High-dose (5 μg/ml) Apixaban incubation was associated with a significantly reduced proliferation in 3 cancer cell lines (OVCAR3, CaCO-2 and LNCaP) and with increased cancer cell mortality in all, except LNCaP, cancer lines. Apoptosis seems to account for the increased mortality. The migration capacity seems to be impaired after high-dose Apixaban incubation in OVCAR3 and CaCO-2 cells. Data on mRNA expression suggest a consistent increase in tumor suppression gene p16 in all cell lines.

**Conclusions:**

Our data suggest that high-dose Apixaban may be able to interfere with cancer cell *in vitro*, reducing proliferation and increasing cancer cell mortality through apoptosis in several cancer cell lines.

## Introduction

Since Trousseau’s observations in 1865, large body of evidence suggests that there is a mutual relation between cancer and blood coagulation: cancer induces a hypercoagulable state that predispose to thrombosis. For more than a century, it is well known that cancer dramatically increases the risk of thrombotic disorders, such as disseminated intravascular coagulation and venous thromboembolism [1,2].

Current guidelines suggest to treat cancer-associated thrombosis with low molecular weight heparin [3]. Active factor X (FXa) inhibitors have recently emerged as alternatives to heparins in cancer patients [4] and, in particular, Apixaban has been suggested to be a possible option for cancer patients with venous thromboembolism [5].

As cancer is therefore linked to hypercoagulability, there have been many efforts in basic research and clinical trials to identify and clarify the possible influence of anticoagulant treatment on tumor development and progression, besides its effects on anticoagulation [6,7]. Studies in animal models have established that the seeding of tumor metastases is a coagulation dependent process [8], and that the non-anticoagulant activity of heparin on metastasis includes the ability to inhibit cell-cell-interaction, to inhibit extracellular matrix protease heparanase, and to inhibit angiogenesis [9]. On the other hand, clinical studies testing the antimetastatic effects of anticoagulant molecules have yielded mixed and no definite results [10–12].

Interestingly, tissue factor pathway inhibitor, a physiological FXa inhibitor, has been described as an inducer of apoptosis in tumor cells, activating caspase, unbalancing anti- and pro-apoptotic protein expression and also preventing metastasis *in vivo* [13]. Recently, amblyomin-X, a Kunitz type FXa inhibitor highly similar to tissue factor pathway inhibitor, has been described as a drug able to reduce the cell viability *in vivo* of several cancer cell lines [6].

Invasion and metastasis are also dependent on specific proteolytic enzymes. Among the protease, the metalloproteases (MMPs) play a critical role in tumor spread, in particular the MMP2 and 9 are the most commonly involved in the extracellular matrix reassembly and tumor progression. Large body of evidence supports the concept of critical role of microenvironment in tumor development [14–17]. Microenvironment is a complex structure constituted by a milieu of molecules accounting proteins as collagens, fibronectin, elastin and complex polysaccharides as proteoglycans and hyaluronan. This area plays an important action in normal tissues, regulating growth factor concentration, nutrients supply and maintaining an intense cross-talk between cells. During the tumor development this carefully organized microenvironment changes dramatically and its functions are completely altered. Moreover, cancer cells secrete procoagulant factors that lead to the activation of platelets and coagulation factors release inflammatory cytokines that affects endothelium [18]. Inflammation is a well-known process in atherosclerosis and vascular diseases, where the endothelial layers are detached from the basal lamina surface in initial damage [19–22]. These anatomical events may further activate the matrix inflammatory milieu.

Correlations between deposition of hyaluronan and malignancy are well documented [23–25]. The hyaluronan around the cancer is usually associated to invasion, cell growth, angiogenesis, lymph angiogenesis, epidermal mesenchymal transition, metastasis, and multidrug resistance [26]. Gene expression of hyaluronan synthase 2 (HAS2) may be therefore considered a marker of malignancy due to hyaluronan properties in induction of cell migration and angiogenesis [27]. MMPs are also related to hyaluronan content in cancer, in fact EMMPRIN (extracellular matrix metalloprotease inducer, CD147) is involved in MMP activation interacting with hyaluronan. In particular MMP9 is involved with CD44 and hyaluronan in a docking process on melanoma cell membranes [28–30].

We analyzed the *in vitro* effects of the direct FXa inhibitor Apixaban on proliferation, mortality, cell migration, expression of key transcription factors (p16, p21 and p53) and HAS2, and MMPs in the following 5 cancer cell lines: OVCAR3 (highly aggressive ovarian cancer cells), MDA MB 231 (highly aggressive breast cancer cells), CaCO-2 (highly aggressive colon cancer), LNCaP (highly aggressive prostate cancer) and U937 (from histiocytic lymphoma).

## Methods

Apixaban was compared with controls (cancer cells without treatment) and with normal cells (two fibroblasts cultures isolated from 2 different tissues–fetal lung and adult dermal fibroblasts-). Apixaban is a highly selective, orally bioavailable, and reversible direct inhibitor of free and clot-bound factor Xa. Apixaban is available as a commercial product named Eliquis^®^, ATC code B01AF02 (WHO). During development it was known as BMS-562247-01. Apixaban for this study was directly supplied by Bristol-Myers Squibb, New Jersey, USA. The cancer cell lines were purchased from ATCC cell culture company (OVCAR3 are ATCC HTB161, MDA MB 231 are ATCC HTB26, CaCO-2 are ATCC HTB37, LNCaP are ATCC CRL1740 and U937 are ATCC CRL1593.2) and lung and dermal normal fibroblasts were purchased from LONZA. In order to assess the drug optimal concentration, all cell cultures, after synchronization (starvation method–overnight incubation with 0.1% fetal bovine serum-), were treated with increasing drug concentrations (0.1 μg/ml, 0,2 μg/ml, 0,5 μg/ml, 1 μg/ml, 5 μg/ml): only the lower concentrations are within the same order of magnitude of measured plasma concentrations after Apixaban per mouth in humans [31]. For *in vitro* experiments each incubation sample was done in triplicate and repeated as many times as reported below for the different experiments. The following time points were considered: 0, 24-hours (h), 48-h, 72-h and 96-h. The comparisons and statistical analysis for evaluating the drug effects were performed using the results obtained at the highest concentration tested, after 96-h.

### Cell cultures

All cells were cultured at 37°C, 5% CO^2^ in specific culture medium: OVCAR3, MDA MB 231, U937 were cultured in DMEM with 10% fetal bovine serum plus bicarbonate, 2mM glutamine and a 100UI penicillin and 100 μg/ml streptomycin; LNCaP were cultured in the same conditions, in RPMI 1640 with 10% fetal bovine serum plus antibiotics, CaCO-2 in the same conditions, in EMEM with 20% fetal bovine serum plus antibiotics.

### Cell proliferation, cytologic aspects and mortality assays

Five cancer cells lines (OVCAR3 cells, MDA MB 231 cells, CaCO-2, LNCaP cells and the U937 line) were added to 2.4 cm diameter wells for proliferation and mortality/vitality assays. Cells were treated with increasing concentration of Apixaban for 24-h, 48-h, 72-h and 96-h with a concentration of the drug ranging from 0.1 μg/ml to 5 μg/ml. For proliferation and mortality/vitality experiments, the number of cells seeded at the beginning of each incubation was carefully checked using 50.000 cells as optimal concentration for our incubation protocol, after a specific experiment in OVCAR3 (S1 Fig). Then we used the same number of cells to be seeded for all the cultures. The need to choice a optimal number of initial cells depends on the scheduled time for evaluation (up to 4 days).

The cells were examined using an inverted light microscope and evaluated for growth characteristics and morphological appearance, and were recorded with a digital camera and computer stored.

In order to exclude potential cell toxicity by Apixaban, cells were analysed by the MTT assay (3-(4,5-dimethylthiazol-2-yl)-2,5-diphenyltetrazolium bromide in all cell lines. A 20 μL volume of 5 μg/mL MTT was added to the cells and the plates incubated for 3 h at 37°C. Afterwards, the medium was discarded, the dark blue formazan crystalline product was dissolved in 100 μL dimethyl sulfoxide, and the absorbance was measured in a microplate reader at 595 nm, in 21 wells.

For proliferation, mortality and vitality, the numbers of cells were counted in 27 culture samples for each point and were assessed by microscope evaluation after Trypan Blue incubation (according to standard procedure). Data are expressed as number of cells (Ncells)/ml.

### Necrosis/Apoptosis assays

Cancer cells of solid tumours were submitted to fluorescence microscopy analysis by using Abcam Kit (ab 176749). After incubation with Apixaban at the concentration of 5 μg/ml, necrosis/apoptosis was evaluated and fluorescent cells were evaluated by fluorescence microscopy (10 different fields analysed for each culture). Briefly, fluorescent analysis shows apoptotic (green, Apopxin Green Indicator), and necrotic cells (red, indicated by 7-AAD staining). Positive controls have been obtained by cell treatment with 1μM staurosporine for 3-h. The fluorescence images of the cells were taken with a fluorescent microscope through the FITC and TRITC channels. Individual images taken from each channel from the same cell population are merged. Live cell cytoplasm labelling dye, CytoCalcein Violet 450 (Ex/Em = 405/450 nm), was used for labelling living cell cytoplasm. Data are expressed as Ncells/field (median values of the 10 fields analysed: green cells are the apoptotic cells; red cells are the necrotic cells). We considered the number of apoptotic cell/fields (Ncell/fields) together with % of apoptotic/total death cells, to compare apoptosis/necrosis in control and Apixaban-incubated cell (5 μg/ml, 96-h).

### Cell migration analysis

A migration test was carried out using cells after scratch test as described in our studies [32,33]. Briefly a confluent cell culture, without and after Apixaban incubation for 96-h, were wounded with sterile tip (time 0) and then the wound healing process was monitored. The healing process was monitored by microscopy measuring the “healing area” at different time (time 0, 6-h, 24-h). Data (expressed as number of cells invading the stretched area after 24-h–Ncell/Area-) are subsequently reported as median of 8 tests each group.

### Metalloprotease analysis

Using zymography, the MMPs activities were tested. After Apixaban incubation for 24-h, 48-h, 72-h and 96-h, the culture medium of each of the 5 cancer cell lines studied was seeded on zymographic gel. Briefly, the cell culture medium was separated by non-denaturizing gel electrophoresis in a gel containing gelatine as substrate copolymerized with polyacrylamide. After incubation, the gels were stained with COMASSIE Blue. The enzyme activity is identified as areas of copolymerized gelatine digested during incubation. The results are shown together standard MMP bands (Calbiochem) and the standard molecular weight bands. This method allows the identification of active inactive form of the enzymes (different bands with different electrophoretic migration).

### Gene expression analysis (p16, p21 p53, HAS1, HAS2 and HAS3; and for U937: CD44, IL1 and IL6; factor X)

In order to evaluate gene expression we performed a quantitative real time RT-PCR. Total RNAs were extracted from cells with TRI reagent (Invitrogen), retro-transcribed using the High Capacity cDNA synthesis kit (Invitrogen), and amplified on an Abi Prism 7000 instrument (Applied Biosystems). Pre-developed Taqman gene expression assays (Invitrogen) were used to quantify transcripts coding for human p16, p21 p53, HAS1, 2 and 3. In U937 cells, CD44 and interleukin (IL)-1 and IL-6 expression were also investigated. β-Actin was used as a housekeeping gene reference. Factor X mRNA synthesis was checked with a specific TaqMan probe from Life Science technologies. The relative gene expression was determined by comparing ΔCt [34] using 2^-ΔΔCt^ method. The comparisons were performed between cancer cells and controls at the same points, in triplicate, each with the appropriate time control (24-h, 48-h and 72-h and 96-h), without and after Apixaban incubation. Data are expressed as fold of increment or decrement.

### Statistical analyses

Data are presented as median and 25–75th percentile range (IQR) for proliferation, mortality assays, apoptosis/necrosis and cell migration, and as means ± standard deviation (SD) for the gene expression analysis experiments. Comparisons between independent measures were performed with the Mann–Whitney U test and unpaired t test when appropriate. For proliferation and mortality tests, calculations were performed at the highest concentration tested at the 96-h time point. Calculations were performed using commercial software (GraphPad Prism version 7.01 for Windows, GraphPad Software, San Diego, CA, USA, www.graphpad.com). A two-sided P<0.05 was retained for statistical significance.

## Results

### Effects of Apixaban on proliferation and mortality in 5 tumor cell lines and two normal fibroblast cultures

**Fig 1:**
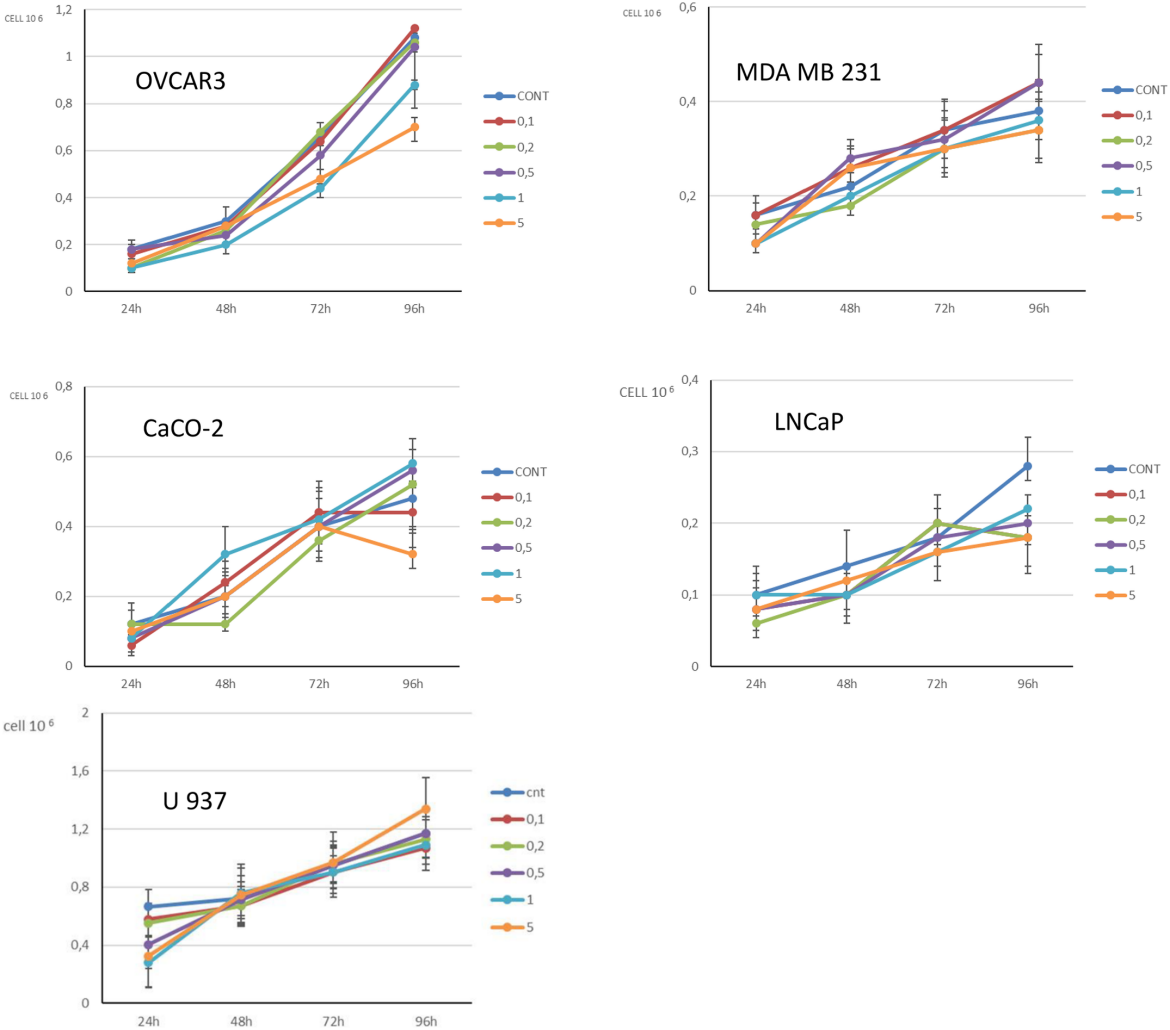
Fig 1 shows proliferation [median (IQR)] in the 5 cancer cell lines analysed. The proliferation is shown for control cancer cells and for cells treated at increasing concentrations of Apixaban (0.1 μg/ml, 0,2 μg/ml, 0,5 μg/ml, 1 μg/ml, 5 μg/ml). The time points considered are: 24-h, 48-h, 72-h. 96-h. Proliferation is expressed as Ncell/ml. At 96-h, a statistically significant difference was observed between control and 5 μg/ml Apixaban treated cells in OVCAR3, CaCO-2, and LNCaP cancer cells (see text).

For all the cultures we tested increasing concentration of Apixaban at the different times considered in order to assess the cell proliferation (Fig 1). The 5 μg/ml concentration (high-dose) of Apixaban at time 96-h resulted in reduced cell proliferation in 3 out of 4 solid tumor cells (OVCAR3: p = 0.0005, Fig 1, panel A; MDA MB 23: p = 0.11, Fig 1, panel B; CaCO-2: p = 0.0001 panel C; LNCaP: p = 0.0001, panel D). For the U937 cells, the proliferation rate was not statistically different between controls and Apixaban treated cells (p = 0.21, Fig 1, panel E) (Table 1).

**Table 1:** Comparisons of cell proliferation between control and Apixaban treated cells in cancer cell lines and normal fibroblasts cultures.

*Cancer cell lines*	Control at 96-h (Ncells x10^6^)	5 μg/ml Apixabanat 96-h (Ncells x10^6^)	P
**OVCAR3**	1.08 (0.90–1.10)	0.70 (0.64–0.74)	p = 0.0005
**MDA MB 231**	0.38 (0.34–0.44)	0.34 (0.32–0.42)	p = 0.11
**CaCO-**	0.48 (0.39–0.62)	0.32 (0.28–0.38)	p = 0.0001
**LNCaP**	0.28 (0.26–0.32)	0.18 (0.14–0.22)	p = 0.0001
**U937**	1.11 (1.08–1.14)	1.34 (1.28–1.40)	p = 0.21
***Normal fibroblasts***			
**- Lung fibroblasts**	0.14 (0.12–0.16)	0.14 (0.14–0.16)	p = 0.88
**- Dermal fibroblasts**	0.08 (0.04–0.10)	0.10 (0.08–0.12)	p = 0.83

Median (IQR) of number of cells x 10^6^

**Fig 2:**
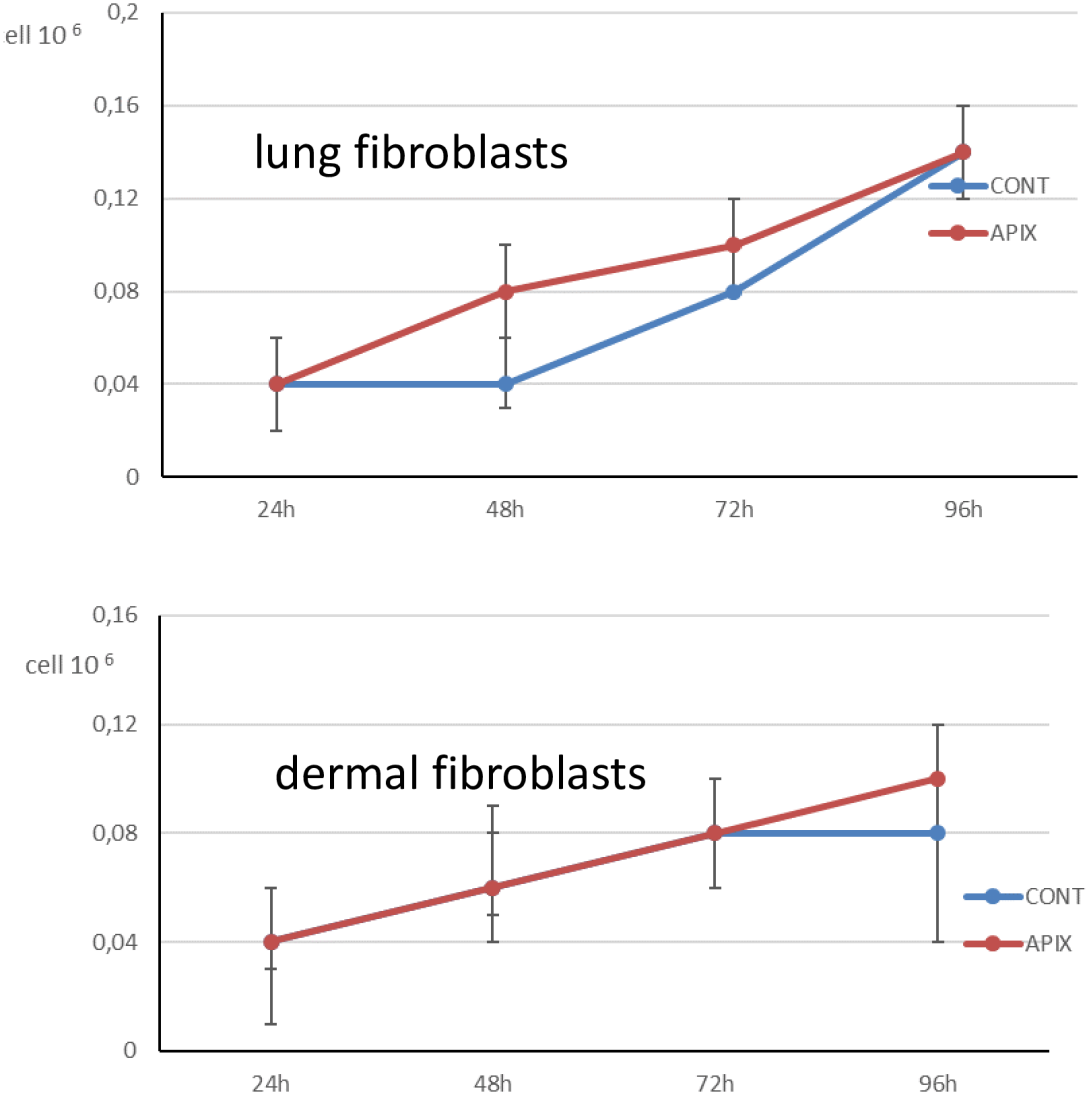
Fig 2 shows proliferation [median (IQR)] in the 2 normal fibroblast culture analysed. The proliferation is shown for control cells and for cells treated at increasing concentrations of Apixaban (0.1 μg/ml, 0,2 μg/ml, 0,5 μg/ml, 1 μg/ml, 5 μg/ml). The time points considered are: 24-h, 48-h, 72-h. 96-h. Proliferation is expressed as Ncell/ml. At 96-h, for 5 μg/ml Apixaban, no statistically significant difference was observed between controls and both fibroblasts cultures (see text).

To confirm a specific effect of Apixaban on solid tumor cell proliferation, we tested this drug at the highest concentration (5 μg/ml) in normal (fetal lung and adult dermal fibroblasts) fibroblasts, after 96-h incubation. In Fig 2 the proliferation rate without and with Apixaban treatment is reported (Lung fibroblast: p = 0.88, Fig 2, panel A. Dermal fibroblasts: p = 0.83, Fig 2, panel B) (Table 1).

**Fig 3:**
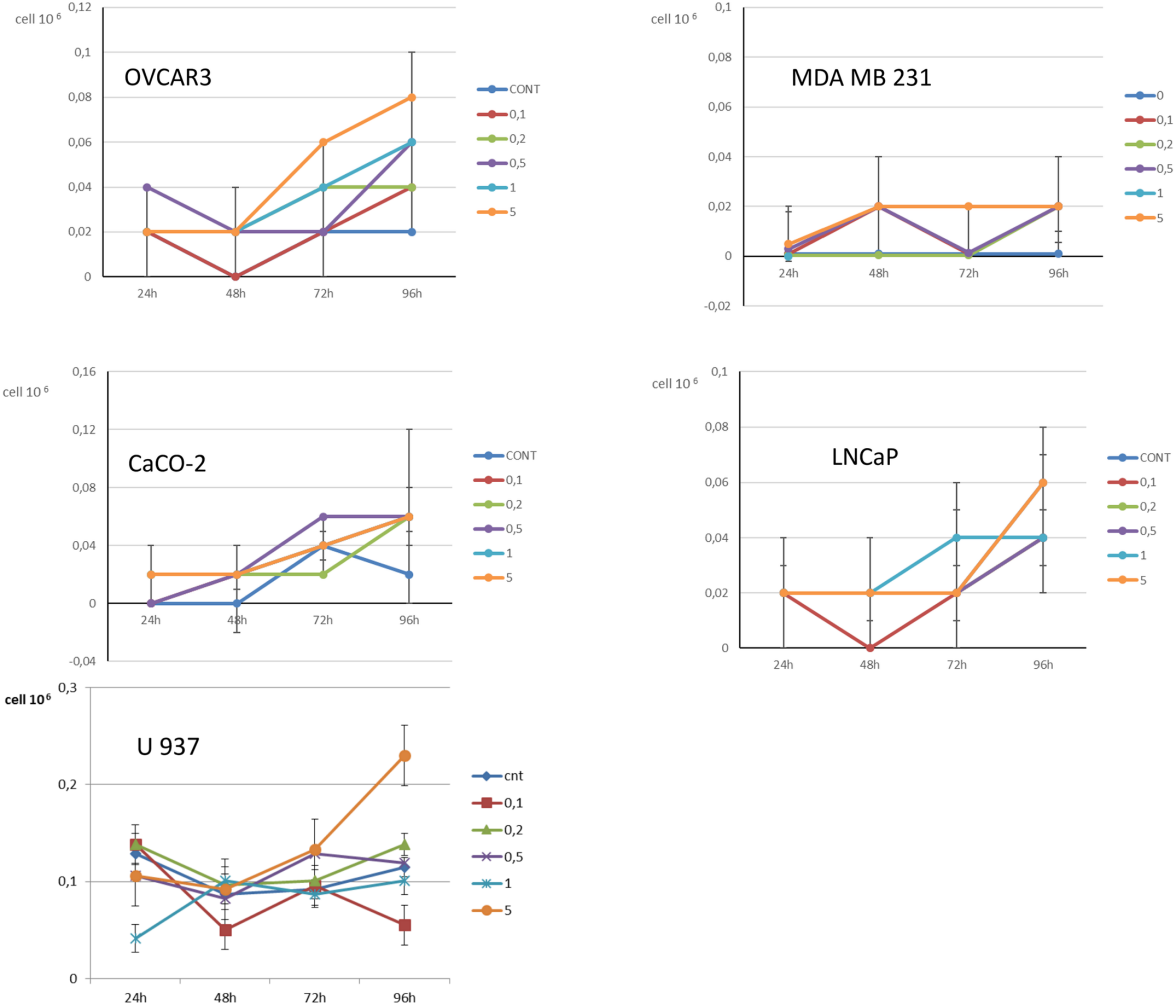
Fig 3 shows mortality [median (IQR)] in the 5 cancer cell lines analysed. The mortality is shown for control cancer cells and for cells treated at increasing concentrations of Apixaban (0.1 μg/ml, 0,2 μg/ml, 0,5 μg/ml, 1 μg/ml, 5 μg/ml). The time points considered are: 24-h, 48-h, 72-h. 96-h. Mortality is expressed as Ncell/ml. At 96-h, a statistically significant difference was observed between control and 5 μg/ml Apixaban treated cells in OVCAR3, MDA MB 231, CaCO-2, and U937 cancer cells (see text).

**Fig 4:**
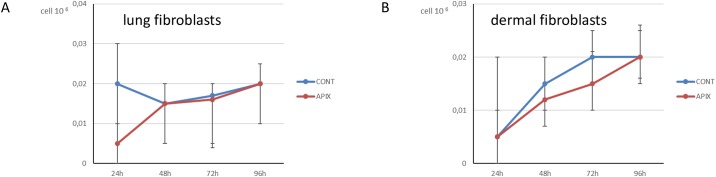
Fig 4 shows mortality [median (IQR)] in the 2 normal fibroblast culture analysed. The mortality is shown for control cancer cells and for cells treated at increasing concentrations of Apixaban (0.1 μg/ml, 0,2 μg/ml, 0,5 μg/ml, 1 μg/ml, 5 μg/ml). The time points considered are: 24-h, 48-h, 72-h. 96-h. Mortality is expressed as Ncell/ml. At 96-h, for 5 μg/ml Apixaban, no statistically significant difference was observed between controls and both fibroblasts cultures (see text).

When cell mortality was considered, Apixaban was associated with an increased cell mortality in 4 out of 5 cancer cell lines at 96-h, for 5 μg/ml, as shown in Fig 3 (OVCAR3: p = 0.029, panel A, MDA MB 231: p = 0.003, panel B; CaCO-2: p = 0.004, panel C, LNCaP: p = 0.20, panel D and U937 cells: p = 0.001 Fig 3, panel E) (Table 2). In Fig 4 is shown mortality in the two fibroblast cultures. Apixaban did not significantly affect mortality in either lung or dermal fibroblasts (p>0.99 for both lung Fig 4, panel A and dermal fibroblasts, Fig 4, panel B) (Table 2).

**Table 2:** Comparisons of cell mortality between control and Apixaban treated cells in cancer cell lines and normal fibroblasts cultures.

*Cancer cell lines*	Control at 96-h (Ncells x10^6^)	5 μg/ml Apixaban at 96-h (Ncells x10^6^)	P
**OVCAR3**	0.02 (0.02–0.04)	0.08 (0.02–0.10)	p = 0.029
**MDA MB 231**	0.0 (0.0–0.05)	0.02 (0.01–0.02)	p = 0.003
**CaCO-2**	0.02 (0.02–0.03)	0.06 (0.06–0.08)	p = 0.004
**LNCaP**	0.06 (0.04–0.06)	0.06 (0.06–0.08)	p = 0.20
**U937**	0.11 (0.10–0.11)	0.19 (0.17–0.21)	p = 0.001
***Normal fibroblasts***			
**Lung fibroblasts**	0.02 (0–0.02)	0.02 (0–0.02)	p>0.99
**Dermal fibroblasts**	0.02 (0–0.02)	0.02 (0.02–0.02)	p>0.99

Median (IQR) of number of cells x 10^6^

When cell vitality was considered, Apixaban was associated with a reduced cell vitality all 5 cancer cell lines (OVCAR3, MDA MB 231, CaCO-2, LNCaP, and U937 cells) (S2 Fig). At 96-h, for 5 μg/ml, the vitality was: OVCAR3: control: 1.06x10^6^ (0.88x10^6^-1.06x10^6^) Ncell/ml, Apixaban: 0.62x10^6^ (0.62x10^6^-0.64x10^6^) Ncell/ml, p = 0.0005, S2 Fig, panel A; MDA MB 231: control: 0.38x10^6^ (0.34x10^6^-0.44x10^6^) Ncell/ml, Apixaban: 0.32x10^6^ (0.31x10^6^-0.40x10^6^) Ncell/ml, p = 0.0001, S2 Fig, panel B; CaCO-2: control: 0.44x10^6^ (0.37x10^6^-0.60x10^6^) Ncell/ml, Apixaban: 0.26x10^6^ (0.21x10^6^-0.30x10^6^) Ncell/ml, p = 0.0001, S2 Fig, panel C; LNCaP: control: 0.24x10^6^ (0.20x10^6^-0.29x10^6^) Ncell/ml, Apixaban: 0.12x10^6^ (0.10x10^6^-0.15x10^6^) Ncell/ml, p<0.0001, S2 Fig, panel D. For the U937 cell cultures, the vitality assay was performed using an automatic cell counter (BIOCELL luna, Logos biosystems, USA) and showed 90% cell vitality in controls after 96-h and 84% in Apixaban treated cell (96-h). In S3 Fig is shown vitality in the two fibroblast cultures. Apixaban did not significantly affect cell vitality in either lung or dermal fibroblasts [lung fibroblasts: control: 0.12x10^6^(0.10x10^6^-0.15x10^6^) Ncell/ml, Apixaban: 0.12x10^6^ (0.10x10^6^-0.14x10^6^) Ncell/ml, p = 0.87, S3 Fig, panel A; dermal fibroblasts: control: 0.06x10^6^ (0.04x10^6^-0.08x10^6^) Ncell/ml, Apixaban: 0.08x10^6^ (0.08x10^6^-0.11x10^6^) Ncell/ml, p = 0.80, S3 Fig, panel B].

### Types of cell death in Apixaban-treated tumor cell lines

**Fig 5:**
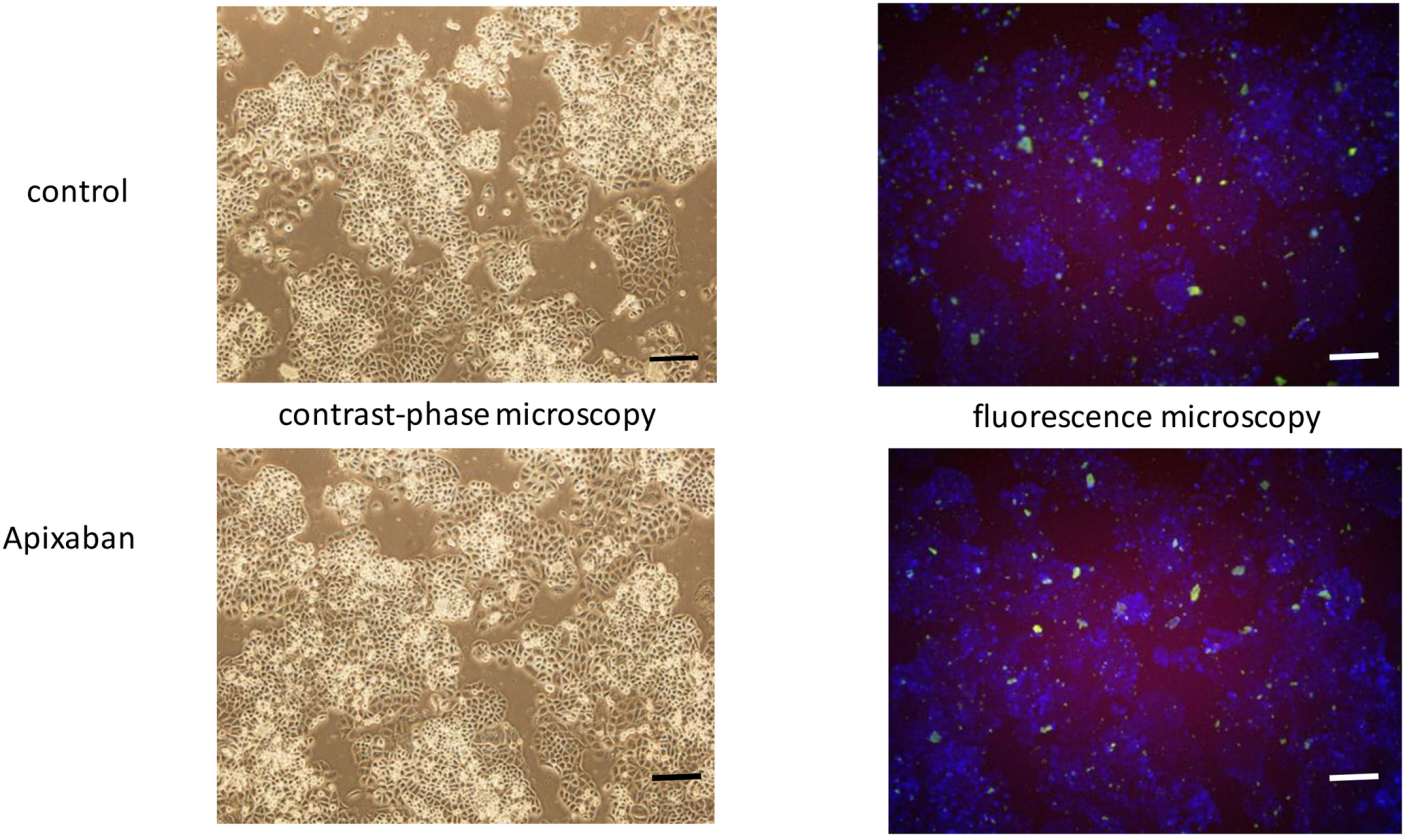
Fig 5 shows an example (control and Apixaban, after 96-h incubation with 5 μg/ml) of apoptosis/necrosis analysis in contrast-phase microscopy fluorescence microscopy for Apixaban and control fluorescence microscopy. The figure shows apoptotic (green, Apopxin green indicator) and necrotic cells (red, indicated by 7-AAD staining). The fluorescence images of the cells were taken with a fluorescence microscope through the FITC (green) and TRITC (red) channels. Individual images taken from each channel from the same cell population were merged in panels B and D. Bars correspond to 100μm; magnification 10x.

To clarify the types of cell death in the increased cell mortality, using fluorescence microscopy analysis we demonstrated that apoptosis is the main modality of cell death as shown for an example in OVCAR3 in Fig 5. Cell mortality was confirmed as increased in Apixaban treated cells, being apoptosis increased after Apixaban in 3/4 solid tumor lines. Results on apoptosis/necrosis of all cell lines (controls, and at 5 μg/ml, at 96-h) are summarized in Table 3.

**Table 3:** Numbers of apoptotic and necrotic cells in solid tumours at 96-h (fluorescence microscopy).

*Cancer cell lines*	Control apoptotic cells	Control necrotic cells	Control % apop/tot	Apixaban apoptotic cells	Apixaban necrotic cells	Apixaban % apopt/tot	p
**OVCAR3**	101 (90–120)	20 (19–20)	83.4%	261 (241–285)	31 (28–32)	90.0%	p = 0.028
**MDA MB 231**	22 (19–26)	9 (8–9)	71.0%	65 (61–71)	10 (9–10)	86.0%	p = 0.029
**CaCO-2**	121 (107–144)	16 (15–16)	88.0%	209 (197–228)	22 (22–25)	90.5%	p = 0.030
**LNCaP**	50 (45–60)	10 (9–10)	83.0%	287 (271–314)	32 (32–36)	87.0%	P = 0.025

Comparisons are made between the numbers of apoptotic cells. Numbers are the median (IQR) of number of cells/fields. The % are the values of apoptotic (apop) cells / total (tot) number of death cells

### Cell migration

**Fig 6:**
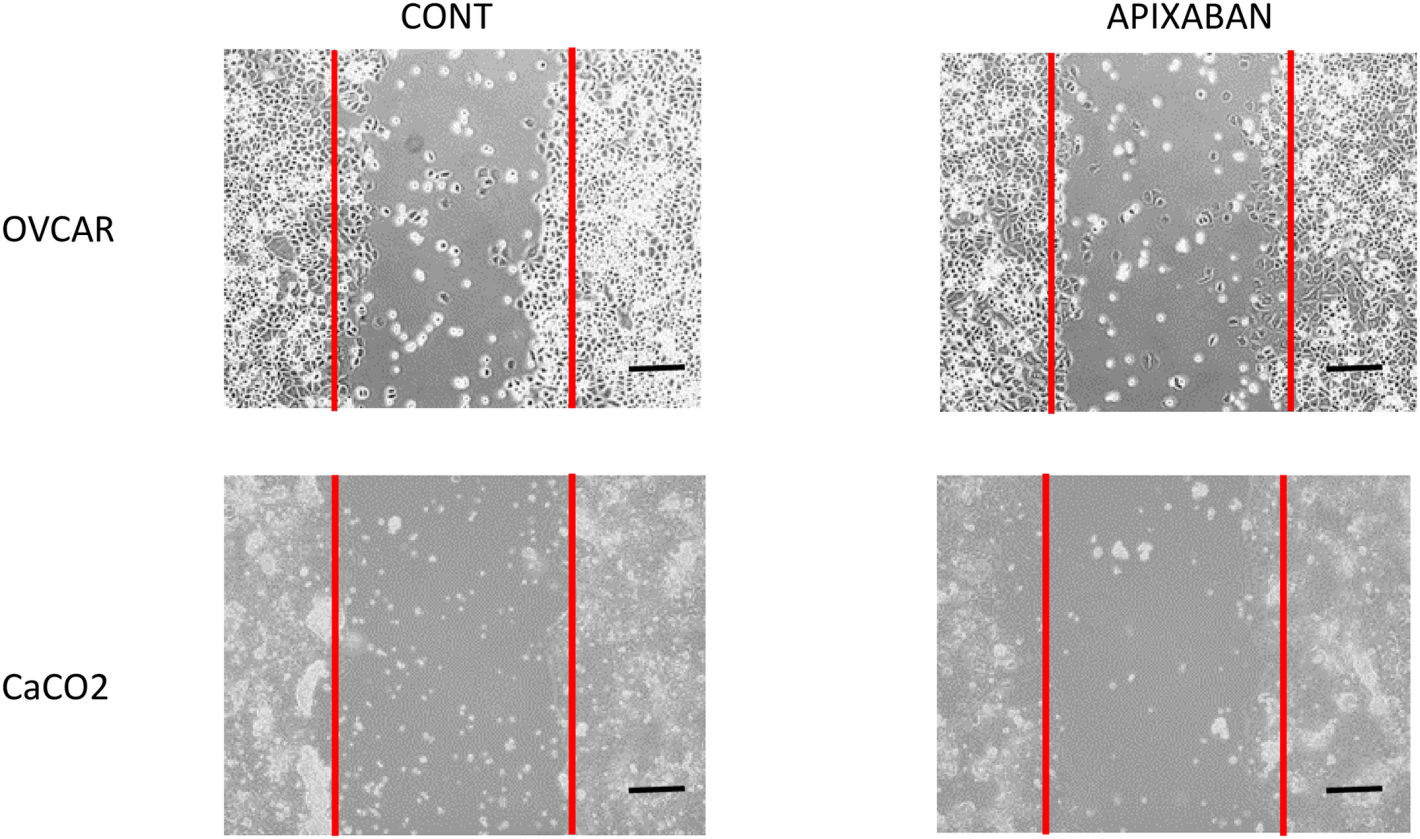
Fig 6 shows an example of cell migration after 24-h from scratch test in controls and cells pre-incubated with Apixaban (96-h, 5μg/ml) in OVCAR3 and CaCO-2 cells. Lines represent the edges of the scratched areas. Bars represents 100μm; magnification 10x.

As cancer migration is a key factor in malignancy spreading, we explored the Apixaban role on this aspect. Incubation with Apixaban 5 μg/ml for 96-h was carried out before scratch. In Table 4 median values of cells after 24-h scratch tests are reported. Apixaban reduced the migratory cell capacity in OVCAR3 and CaCO-2 cell lines. Fig 6 shows an example in OVCAR3 and CaCO-2 of a scratch test in controls and after 24-h after stretch.

**Table 4:** Migratory capacity measured 24-h after stretch tests in controls and Apixaban cancer cell cultures.

*Cancer cell lines*	Control, Ncell/Area over the edge at 24h	Apixaban, Ncell/Area over the edge at 24h	p
**OVCAR3**	311 (296–328)	186 (183–195)	p = 0.002
**MDA MB 231**	130 (127–139)	133 (125–140)	p>0.99
**CaCO-2**	250 (235–250)	174 (159–187)	p = 0.043
**LNCaP**	82 (77–86)	89 (83–96)	p = 0.33

Comparisons are made between the numbers of cells invading the stretched area (Ncell/Area) after 24-h. Numbers are the median (IQR) of number of cells/fields.

### Metalloprotease analysis

**Fig 7:**
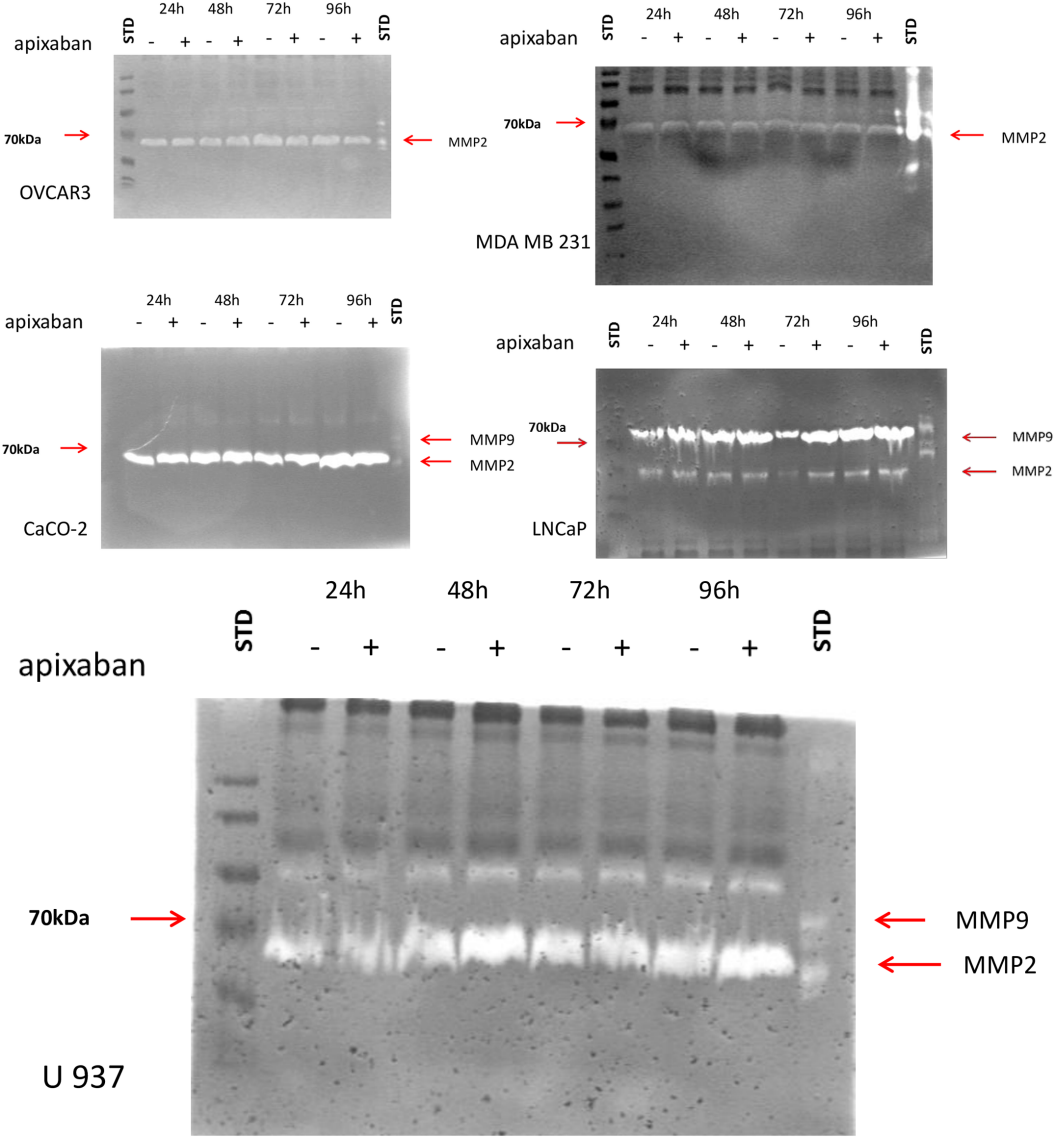
Fig 7 shows the zymographies of metalloproteases (MMP) produced by the 5 cancer cell lines analysed in control cells (proteases derived by control cells are shown in lanes marked with a “-”sign; the proteases derived by Apixaban 5 μg/ml treated cells are shown in lanes marked with a “+” sign). The time points considered are: 24-h, 48-h, 72-h. 96-h. Standard lanes at the left of each figure are Molecular weight standards and at the right of each figure are the commercial MMP 2 and 9 standards. At all the time points considered, MMP2 is detectable in all cell lines (control and treated cells) whereas MMP9 is detectable in LNCaP and in U937. The bands are expressed as zymogens and no evidence of activated bands are detectable.

Cancer migration is strongly dependent on the microenvironment and matrix degradation; therefore, we measured the MMPs activity produced from the cancer cell lines in the presence of Apixaban. Fig 7 shows the enzyme activity of MMPs before and during Apixaban.

All the solid tumors, and histiocytic lymphoma cells produce MMP2 as inactive form. LNCaP cells also produce MMP9. No activated band can be detected indicating that Apixaban is not able to activate the MMPs produced by cancer cells. Fibroblast produced MMP2 as inactive form and no change was observed after Apixaban incubation.

### Effect of Apixaban on gene expression

Gene expression of proteins involved in tumor biology was investigated.

#### p16, p21 p53

At baseline, in U937 cells, p16 and p53 mRNAs were not detectable; therefore, Apixaban was not tested for p16 and p53 in these cells.

The p16 expression at 96-h resulted significantly increased in all cell lines OVCAR3, MDA MB 231, CaCO-2, and LNCaP (Table 5). Moreover, most of the time points showed a significant difference in control and treated cells.

**Table 5:** mRNA gene expression (2^-ΔΔCT^) in cell cultures after Apixaban (5 μg/ml) incubation.

*mRNA*		*OVCAR3*	*MDA MB 231*	*CaCO-2*	*LNCaP*	*U937*
**p16**	T 24-h	0.88±0.02**	0.42±0.03**	1.12±0.00***	1.24±0.02*	NA
	T 48-h	1.02±0.00*	1.55±0.05*	1.13±0.08	1.01±0.00*	NA
	T 72-h	1.26±0.01*	1.56±0.10	1.00±0.01	0.81±0.01**	NA
	T 96-h	1.14±0.00**	1.56±0.10**	1.38±0.04***	1.12±0.04***	NA
**p21**	T 24-h	0.91±0.03*	1.12±0.02	0.86±0.11	1.08±0.11*	1.20±0.13**
	T 48-h	0.76±0.00**	1.01±0.01	1.00±0.01	0.98±0.06	1.07±0.18
	T 72-h	0.88±0.01*	1.05±0,01	1.05±0.20	0.84±0.14	1.04±0.18
	T 96-h	1.08±0.01	0.88±0,01	0.95±0.03**	0.78±0.06*	1.14±0.05
**p53**	T 24-h	0.95±0.07	1.23±0.00*	0.95±0.09	0.97±0.08**	NA
	T 48-h	0.79±0.00***	0.87±0.05*	1.18±0.03**	1.04±0.08*	NA
	T 72-h	0.86±0.03	1.07±0.04	0.72±0.30*	0.84±0.38*	NA
	T 96-h	0.89±0.01*	0.89±0.09	0.92±0.39	0.73±0.17	NA
**HAS2**	T 24-h	1.91±0.03***	0.98±0.03***	1.13±0.06***	NA	NA
	T 48-h	0.72±0.04***	0.86±0.18	0.89±0.05	NA	NA
	T 72-h	0.60±0.01***	1.02±0.03	0.87±0.05***	NA	NA
	T 96-h	2.70±0.03***	0.88±0.00*	1.11±0.10	NA	NA

Comparisons are made between expression of mRNAs, between cancer cell cultures and Apixaban treated cultures, each compared with its control.

* indicates p<0.05;

** indicates p<0.01,

*** indicates p<0.001;

NA indicates not applicable.

SE values are indicated.

As for p21, different responses were observed in the cancer lines, and at the different time points being the mRNA decreased at 96-h for CaCO-2, LNCaP. In U937 cells which did not express p16, p 21 was increased at 24-h, tended to be increased at 48-h (p = 0.07) and did not result significantly different at 96-h (Table 5).

In OVCAR3, p53, resulted down-regulated since the 48-h time point (p<0.001), tended to be reduced at 72-h (p = 0.08) and was statistically reduced at 96-h whereas in MDA MB 231, CaCO-2 and LNCaP, p53 did not showed a linear trend and was not statistically different at 96-h (Table 5).

#### HAS2

HAS2 was expressed in OVCAR3, MDA MB 231 and CaCO-2 lines and represents the main hyaluronan synthase. HAS1 was not detectable whereas HAS3 was only present in OVCAR3 cells at a lower amount therefore we tested Apixaban for HAS2 expression in the 3 cancer lines described above. The HAS2 was differently expressed in the different lines, and at the different time points analyzed, being increased at 96-h in OVCAR3 after a reduction observed at 48 and 76-h, reduced at 96-h in MDA MB 231 and unchanged in CaCO-2 (Table 5).

#### CD44 and IL1 in U937 cells

Since this line of histiocytic lymphoma does not express HAS2, the hyaluronan receptor CD44 was investigated, as this receptor is commonly expressed. To explore the inflammatory response in lymphoid lineage cells that are known to produce inflammatory cytokines, the mRNA for IL1 and IL6 were tested in controls, and in the presence of Apixaban (as for IL1 which was found expressed in control cells whereas IL6 expression was not detectable). In these cells CD44 and IL1 at 96-h in Apixaban treated cells were reduced (the values using 2^-ΔΔCT^ method were 0.85 p<0.05 with respect to its control, and 0.73 p<0.05 with respect to its control, respectively).

#### Factor X

The amount of factor X mRNA is completely absent in cancer cells. We detected very low amount of factor X mRNA in the fibroblasts.

## Discussion

To the best of our knowledge, we reported first data on the possible effects of high-dose Apixaban on various cancer type lineages. We investigated the effect of this direct FXa inhibitor on proliferation, mortality, cell migration, production of MMPs and expression of oncogenes and HAS in OVCAR3 (ovarian cancer cells), MDA MB 231 (breast cancer cells), CaCO-2 (colon cancer), LNCaP (prostate cancer) and U937 (histiocytic lymphoma), as the procoagulant activity may differ in different cancer cell lines [35–36]. Our results suggest that high-dose (5 μg/ml) of Apixaban incubation may be associated with reduced proliferation in 3 out of 5 cancer cell lines (OVCAR3, CaCO-2 and LNCaP). Moreover, most of the tumor lines showed a possible increased cell mortality during the time points up to 4 days incubation, that was significant at 96-h for 4/5 cancer lines OVCAR3, MDA MB 231, CaCO-2, and U937 cells. No effects on proliferation and mortality, potentially related to toxic drug effect, were observed in both normal fibroblasts cultures upon Apixaban incubation. The increased cancer cell mortality during high-dose Apixaban incubation was shown by fluorescence microscopy analysis, suggesting that increased apoptosis may explain this effect.

To date, although the hemostatic system is involved in the mechanisms of cancer growth and metastasis, the molecular events underlying the interferences of anticoagulant drugs with tumor biology are largely unknown [37]. Sporadic reports on antiproliferative action of heparin and low molecular weight heparins on cancer cells have been published [38–41] whereas no data exist on the mechanisms potentially relating malignancy and direct FXa inhibitors. Moreover, although all heparins caused a dose-dependent reduction in cellular growth, when different oligosaccharides of enoxaparin obtained through ion-exchange chromatography were used, only two oligosaccharides showed distinctive anti-proliferative effects while the majority of these oligosaccharides stimulated proliferation [40], indicating specific effects of slightly different molecules.

In our study, the migratory capacity, a key step in the metastatic cascade, seems to be reduced in OVCAR3 and CaCO-2 tumor cells after Apixaban incubation. Activation of protease-activated receptors has been related most to increased cell migration [42–46]. It has been suggested that protease-activated receptor-2 is the endogenous receptor for upstream coagulant protease Xa and VIIa signaling in breast cancer cells, and that FXa and tumor-generated proteases may activate protease-activated receptors to promote cell invasion and metastasis [42]. Moreover, activation of the mammalian target of rapamycin pathway, which is probably mediated by protease-activated receptors, was associated with enhanced cell migration, and specific inhibition of this pathway with rapamycin markedly decreased cell migration induced by formation of Tissue Factor-Factor VIIa-FXa complex [43]. Protein-activated-receptor-2-specific antibodies fully attenuated Tissue Factor-Factor VIIa-induced IL-8 expression [44]. In our experiments in histiocytic lymphoma, the inflammatory response, as expressed by mRNA for IL1 resulted down-regulated by Apixaban.

Previously, Nadroparin has been suggested to reduce the invasive, migratory, and adhesive ability of human lung adenocarcinoma A549 cells by down-regulating the expression of integrin β1 and β3 as well as matrix MMP2 and 9 and by restraining the actin cytoskeleton reorganization [47]. In our study, MMP2 (and MMP9 for LNCaP) were produced by cell tumors in inactive forms and Apixaban did not activate the MMPs produced by cancer cells. MMP2 and MMP9 are "FXa-like" protease. If any effect of Apixaban exists on a cancer cell membrane "FXa-like" protease, this is not mediated by MMP2 and MMP9. Despite the observation of inactive MMPs in culture medium, we cannot infer about the activity status in solid tumors since it has been reported that *in vitro* and *in vivo* regulation and secretion of MMP-2 and MMP-9 may differ [48].

In the gene expression analysis, p16 (INK4a) expression seems to be increased upon high-dose Apixaban incubation. This behavior was apparently shared by all the cancer lines tested, which consistently showed p16 increase at the different time point tested. In cancer and precancerous lesions, p16 is considered a tumor suppressor gene and pushes cells to enter senescence, an irreversible cell-cycle arrest and loss of p16 is one of the most frequent events in human tumors allowing precancerous lesions to bypass senescence [49–50]. The p21 protein acts as a regulator of cell cycle progression and contributes to mediate cellular senescence. This gene expression is controlled by p53 in response to stress stimuli [51–53]. As for p21, different responses were observed in the cancer lines after Apixaban treatment, being the mRNA decreased at 96-h for CACO-2 and LNCaP. In previous studies, Nadroparin inhibited proliferation of A549 cells but p21 mRNA expression was not modified [40]. Upon DNA damage or other stresses p53 is activated and induces a cell cycle arrest to allow both repair and survival of the cell or apoptosis. Although classically associated with apoptosis, this protein has been suggested to display also antiapoptotic effects. In fact, p53 protects cells against UV- and cisplatin-induced apoptosis in a manner dependent on transcription-coupled DNA repair [54], therefore its effect seems to be the result of an intricate network of signals and molecular interactions [55]. Moreover it has been recently reported in 162 tumors in pancreatic cancer patients that high proliferation of tumors and strong and consistent nuclear p53 expression by tumor cells (immunohistochemistry) is associated with a worse disease-free survival and overall survival in the overall study population [56]. In our study p53 mRNA resulted down-regulated in OVCAR3 and tended to be reduced in all cell lines.

Results on HAS2 in solid tumors cultures were not conclusive in our study, showing different patterns at the different timing across the cell lines. At 96-h HAS2 was markedly increased in OVCAR3 and reduced in MDA MB 231 after treatment. We previously reported that cancer cells may express increased HAS activity upon stressful stimuli [57]. Increased stressful cellular conditions in OVCAR3 cells may account for the increased expression of HAS2 observed in this cell line. However, in histiocytic lymphoma that constitutionally does not express HAS2, when CD44 mRNA was tested upon treatment with Apixaban to explore the hyaluronan receptor expression, CD44 was reduced by drug treatment. Hyaluronan can bind different cell receptors, as CD44, CD168/RHAMM, lymphatic vessel endothelial receptor, layilin and Toll-Like Receptors. The interaction with receptors starts the intracellular signaling, for example CD44 interacting with hyaluronan induces the ERK1/2 phosphorylation and its nuclear translocation and, depending on the cell types, promotes cell aggregation, migration, proliferation and angiogenesis. The cells after hyaluronan stimulation throughout CD44 produce TGFbeta and bFGF and in ovarian cancer model CD44 interacts with Her2 and ErbB2 in presence of hyaluronan inducing cell growth. Moreover, the large size hyaluronan can interfere with cell apoptosis using CD44 or Toll like receptor4 [32].

Our study has some limitations. First, many of the effects of Apixaban were detected at a concentration of 5 μg/ml, at least ten times higher than that measured in the plasma after intake of Apixaban in humans for clinical use [31,58]. As the on- or off-target effect of Apixaban should be broader since the drug seems to affect proliferation and migration in a FXa-independent manner (as FXa mRNA is not expressed), there may be serious side effects if used *in vivo*. Second, the experiments were performed in a cell culture milieu that contained 10% bovine serum albumin, which may contain traces of activated clotting factors that could promote cell proliferation. However, data sheet does not show possible contaminants from clotting factors at the mass spectrometry analysis. Moreover, we tested different concentration of fetal bovine serum and the differences of cell growth are very small and statistically irrelevant at the time of incubation. Third, as no data were available in the literature on the effect of apixaban on cell proliferation, mortality, cell migration, gene expression and matrix metalloproteinase in cancer cell lines, we performed these preliminary experiments to provide first evidence future research programs. Next studies should investigate possible underlying mechanisms, e.g. presence of "FXa-like" protease on cancer cell membrane, the inhibition of which changes the biological process of cancer cells, and other possible effects, e.g. cell migration. Moreover, next experiments should be performed with cells from a tissue of patients with the different stages/hormonal status of disease, and with experimental animals that can get this drug orally.

In conclusion, high-dose (5 μg/ml) Apixaban seems to reduce cancer cell proliferation and to increase cancer cell mortality through apoptosis in different cancer cell lines *in vitro*. Results on gene expression of tumor suppressor genes such as p16 (increased) may be consistent with increased cancer cell mortality upon Apixaban cell treatment.

The reduced migration capacity of cancer cells after Apixaban incubation may indicate a reduced malignancy potential in some cancer cell lines, though our results on MMPs and HAS2 do not clarify the mechanisms potentially associated with a reduced invasion capacity.

## Supporting information

S1 Fig S1 Fig shows results of experiments carried out in order to set the optimal cell concentration to be used for the subsequent experiments. Median (IQR) of proliferation in OVCAR3 using 25000, 50000 and 100000 cells seeded at time 0.
(TIFF)

S2 Fig S2 Fig shows cell vitality [median (IQR)] in the 4 solid cancer cell lines analyzed (Panel A: OVCAR3; Panel B: MDA MB 231; Panel C: CaCO-2; Panel D: LNCaP; Panel E: U 937). The vitality is shown for control cancer cells and for cells treated at increasing concentrations of Apixaban (0.1 μg/ml, 0,2 μg/ml, 0,5 μg/ml, 1 μg/ml, 5 μg/ml). The time points considered are: 24-h, 48-h, 72-h. 96-h. Proliferation is expressed as Ncell/ml. At 96-h, a statistically significant difference was observed between control and 5 μg/ml Apixaban treated in all the solid tumour cell lines. (see text).
(TIFF)

S3 Fig S3 Fig shows vitality [median (IQR)] in the 2 normal fibroblast culture analysed (Panel A: lung fibroblasts; Panel B: dermal fibroblasts). The vitality is shown for control cells and for cells treated at increasing concentrations of Apixaban (0.1 μg/ml, 0,2 μg/ml, 0,5 μg/ml, 1 μg/ml, 5 μg/ml). The time points considered are: 24-h, 48-h, 72-h. 96-h. Vitality is expressed as Ncell/ml. At 96-h, for 5 μg/ml Apixaban, no statistically significant difference was observed between controls and both fibroblasts cultures (see text).
(TIFF)
