# Supplementary figures and images for: In vitro effects of Apixaban on 5 different cancer cell lines
Source: PLoS One. 2017 Oct 12;12(10):e0185035. doi: 10.1371/journal.pone.0185035 (PMC5638249; doi:10.1371/journal.pone.0185035)

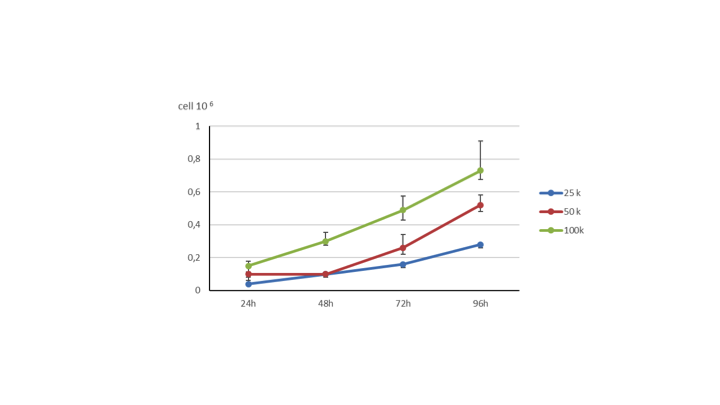

Supplement: S1 Fig — Median (IQR) of proliferation in OVCAR3 using 25000, 50000 and 100000 cells seeded at time 0. (TIFF) [file pone.0185035.s001.tiff]

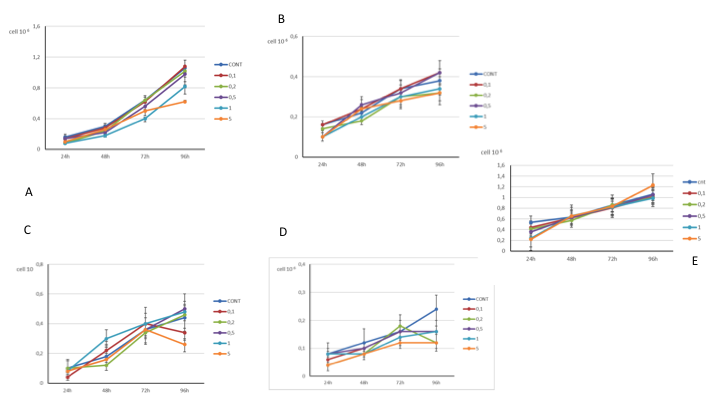

Supplement: S2 Fig — The vitality is shown for control cancer cells and for cells treated at increasing concentrations of Apixaban (0.1 μg/ml, 0,2 μg/ml, 0,5 μg/ml, 1 μg/ml, 5 μg/ml). The time points considered are: 24-h, 48-h, 72-h. 96-h. Proliferation is expressed as Ncell/ml. At 96-h, a statistically significant difference was observed between control and 5 μg/ml Apixaban treated in all the solid tumour cell lines. (see text). (TIFF) [file pone.0185035.s002.tiff]

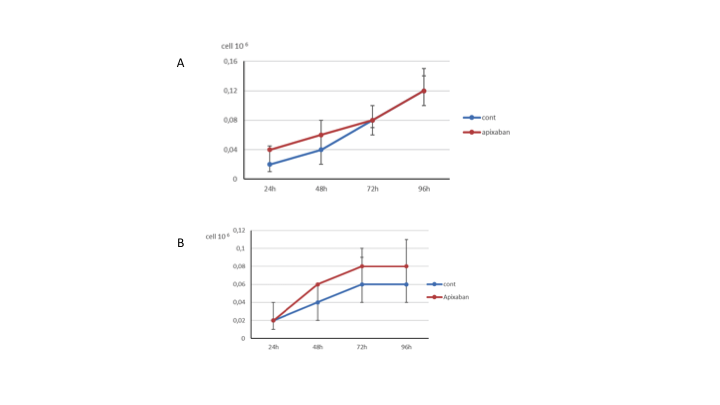

Supplement: S3 Fig — The vitality is shown for control cells and for cells treated at increasing concentrations of Apixaban (0.1 μg/ml, 0,2 μg/ml, 0,5 μg/ml, 1 μg/ml, 5 μg/ml). The time points considered are: 24-h, 48-h, 72-h. 96-h. Vitality is expressed as Ncell/ml. At 96-h, for 5 μg/ml Apixaban, no statistically significant difference was observed between controls and both fibroblasts cultures (see text). (TIFF) [file pone.0185035.s003.tiff]
